# Thoracentesis

**Published:** 1875-02-15

**Authors:** 


### THORACENTESIS.

*From The Doctor, January 1, 1875.*

IN an able communication on pleuritic effusion generally, Dr. J. R. Wardell, of Tunbridge Wells, thus states the conditions which may be regarded as the morbid states, and the positive and negative signs demanding thoracentesis:

1. In all cases in which inspection and the physical signs give evidence of a large quantity of fluid, when there are symptoms of compression of the lung, and there is manifest cardiac displacement.

2. When there are urgent dyspnœa, an irregular pulse, and threatening of orthopnœa.

3. When the affected side is smooth and rounded, and the intercostal spaces are effaced or protrude; when measurement proves bulging; when the dullness in the chest is complete, or demarcated and absolute; when there is abolition of tactile fremitus; when there are bronchophonic voice, tubular breathing, and absence of breath-sound; when the patient can only lie on one side, or in diagonal position; and when there is the Hippocratic sign of succussion.

4. When the exploratory needle proves the fluid to be purulent.

5. If the heart be pushed from its normal situation, and the apex be substernal or beyond the right sternal edge, or if it be thrust toward the left hypochondrium, or if it be lost; when it becomes presumptive that the organ has been driven inwards and backwards; and when on the one side the liver depends abnormally into the abdomen, and when on the other side the relaxed and down-pressed diaphragm so displaces the spleen that its free edge can be *felt*.

6. When half the thoracic cavity is filled, and a month or so shows no proof of absorption, the longer the delay the less are the chances of expansion.

7. In those exceptional cases of double pleurisy, when both cavities become half-filled with effusion, and dyspnœa shows the lung space to be dangerously encroached upon.

8. In pulmonary phthisis, when the accumulation of serous or sero-purulent secretion causes distress, and when the other lung assumes the symptoms of bronchitis or pneumonia, the operation should at once be performed.

9. In mechanical hydrothorax it may be had recourse to, though with no object to cure, but with merely a view for a time to prolong life, and to aid the action of medicinal remedies.

10. In children, whose chest walls are thin, and in whom the white tissues are more developed and confer greater resiliency to the thoracic parietes, and whenever there are certain evidences of fluid, it should without delay be evacuated.

11. In hydropneumothorax it may be generally with safety and benefit employed.

12. Pointing externally should never be waited for.

13. Under certain circumstances repeated tappings are required.

Dr. H. F. A. Goodrich, of Bath, relates a case of acute pleuritic effusion, steadily advancing until the twelfth day, on which 45 oz. of fluid were removed, when the absorption became active and recovery was attained by the fortieth day. This case he contrasts with another which was treated without operation; but although the issue of the case was satisfactory, inasmuch as the patient recovered, Dr. Goodrich thinks that the comparative disadvantages of its treatment can hardly be overlooked, viz.:

1. The uncertainty of success, which uncertainty, it is maintained, cannot in anything like equal degree, if at all, be predicated of thoracentesis practiced with due precautions, and with the improved instruments; practiced, in short, as it now is.

2. The prolongation of the period of danger here represented by the four or five days following the admission of the patient. Sudden death was then a liability, and the youth of the subject would afford no adequate protection against it.

3. The length of time under treatment, and the comparative slowness of convalescence.

Dr. D. M. Williams, of the Liverpool Consumption Hospital, is not so disposed as the previous writers to early operation, thinking that serum will re-accumulate and probably become purulent. He relates a case in which an operation was performed, and the pus evacuated was found to be fœtid, although he believed no fistulous communication existed between the lung and the pleural cavity. After the evacuation of the pus a rapid improvement took place.

Mr. William Date, of Crewkerne, tapped a patient who had all the signs of simple pleuritic effusion, and was somewhat surprised to find that pus made its exit. Although the aspirator was employed, the pus that re-accumulated became offensive. The patient was tapped several times, with great relief on each occasion, but subsequently sank, as so many cases of empyema do. No post-mortem was permitted.

In the *Journal de Medecine et de Chirurgie* for December are reported some observations recently made by M. Peter, of Paris, in his clinique at the Hopital Saint-Antoine. M. Peter remarked that puncture with the trocar in purulent pleurisy is often followed by the formation of a purulent fistula. It was important not to be misled on this point, and every time a purulent pleurisy is punctured the patient and his friends should be forewarned. Immediately after the puncture, the wound and the wall of the thorax appeared to close easily. At the end of a certain time, however, the little wound opens again, and evacuates a certain quantity of pus; then it closes again, to open later on; and a permanent fistula is gradually formed. Unless the patient has been warned of the probability of this event happening, he will probably accuse the physician of unskillful management, whilst it is really a consequence of the nature of the disease.

---

